# Neferine Attenuates the Protein Level and Toxicity of Mutant Huntingtin in PC-12 Cells via Induction of Autophagy

**DOI:** 10.3390/molecules20033496

**Published:** 2015-02-18

**Authors:** Vincent Kam Wai Wong, An Guo Wu, Jing Rong Wang, Liang Liu, Betty Yuen-Kwan Law

**Affiliations:** State Key Laboratory of Quality Research in Chinese Medicine, Macau University of Science and Technology, Macau, China; E-Mails: kawwong@must.edu.mo (V.K.W.W.); wag1114@foxmail.com (A.G.W.); jrwang@must.edu.mo (J.R.W.)

**Keywords:** autophagy, *Nelumbo nucifera*, neferine, AMPK, mTOR, α-synuclein, huntingtin, PC-12, bioactivity, natural products, mechanism of action

## Abstract

Mutant huntingtin aggregation is highly associated with the pathogenesis of Huntington’s disease, an adult-onset autosomal dominant disorder, which leads to a loss of motor control and decline in cognitive function. Recent literature has revealed the protective role of autophagy in neurodegenerative diseases through degradation of mutant toxic proteins, including huntingtin or α-synuclein. Through the GFP-LC3 autophagy detection platform, we have identified neferine, isolated from the lotus seed embryo of *Nelumbo nucifera*, which is able to induce autophagy through an AMPK-mTOR-dependent pathway. Furthermore, by overexpressing *huntingtin* with 74 CAG repeats (EGFP-*HTT* 74) in PC-12 cells, neferine reduces both the protein level and toxicity of mutant huntingtin through an autophagy-related gene 7 (Atg7)-dependent mechanism. With the variety of novel active compounds present in medicinal herbs, our current study suggests the possible protective mechanism of an autophagy inducer isolated from Chinese herbal medicine, which is crucial for its further development into a potential therapeutic agent for neurodegenerative disorders in the future.

## 1. Introduction

Autophagy refers to the lysosomal degradation of damaged or superfluous organelles and proteins to recycle cellular constituents and nutrients for maintaining cellular homeostasis. Autophagy starts with the formation and expansion of an isolated membrane, which can elongate and form a double-membrane vesicle called the autophagosome. All engulfed cytoplasmic materials are then sequestered inside the autophagosome, which subsequently fuses with the lysosome for degradation [1].

While autophagy is constitutively active at a low basal level, it is also responsible for regulating normal neuronal homeostasis [2]. It has been reported that defects in autophagy regulation, such as SQSTM1(p62) mutations [3], autophagy-related gene (Atg) 9 mislocation [4] or mutant huntingtin-mediated impairment of Beclin 1-mediated protein turnover [5,6], are associated with neurodegenerative disorders, including amyotrophic lateral sclerosis (ALS), Parkinson’s disease (PD) and Huntington’s disease (HD), respectively. In mouse models with defective autophagy function, neurodegeneration and protein inclusion accumulation were reported [7]. This suggests the essential role of autophagy in maintaining healthy neurons and modulating neurodegenerative disorders through effective protein quality control. In fact, regular protein quality control for neurons is important, because mutant proteins and damaged organelles cannot be reduced through cell division in neurons, and therefore, these malfunctioned structures must be identified and cleared through autophagic degradation before they accumulate and lead to neurotoxicity [2,8].

With the protective effects of lowering the level of toxic protein aggregates, autophagy has recently become an attractive therapeutic target for neurodegenerative disorders. Huntington disease, a neurodegenerative disease characterized by progressive motor dysfunction and dementia [9], is caused by a larger than 35 CAG trinucleotide repeat expansion, which results in a long mutant polyglutamine tract in the huntingtin (HTT) protein [10]. These polyglutamine expansions are highly associated with cytotoxicity and aggregate formation [11]. To this end, the identification of compounds that enhance autophagy in HD is highly desirable. Recently, a United States Food and Drug Administration-approved drug, rilmenidine, was reported for its ability to induce autophagy and attenuate the toxicity of mutant huntingtin in a mouse model of HD [9]. Another neuroprotective dietary flavonoid, fisetin, can induce autophagic cell death through the mTOR pathway [12]. Furthermore, rapamycin, an inducer of mammalian target of rapamycin (mTOR)-dependent autophagy, is effective at increasing the autophagic clearance of mutant HTT fragments *in vivo* [6,13]. However, rapamycin can affect protein synthesis and cell proliferation [1,14]; fisetin has the disadvantage of high effective concentration, low lipophilicity and poor bioavailability [12]. Therefore, further identification of chemicals that can enhance the autophagic clearance of mutant aggregate-prone proteins with fewer side effects would be desirable.

In our current study, we have identified a small-molecule autophagy inducer, neferine, a bisbenzylisoquinoline alkaloid from the traditional Chinese medicinal plant, *Nelumbo nucifera* Gaertn, as a potential neuro-protective agent. According to Shenong’s report in the Liang Dynasty of China, *Nelumbo nucifera* is usually prescribed for relieving anxiety and anti-aging in traditional Chinese medicine. Besides, it is commonly used as an ingredient of tea or soup due to its non-toxic nature. However, the protective mechanisms by which *Nelumbo nucifera* benefits human health remain unclear. In this study, we set out to investigate the novel protective function of neferine in modulating HD by cellular assays. We herein present evidence that neferine lowered the protein level and toxicity of mutant HTT in PC-12 cells through an autophagy-related gene 7 (Atg7)-dependent pathway. Taken together, our results report for the first time the neuro-protective function of neferine at the cellular level, which may provide important information for its further development into a neuro-protective agent.

## 2. Results and Discussion

### 2.1. Neferine Induces Autophagy in PC-12 Cells

**Figure 1:**
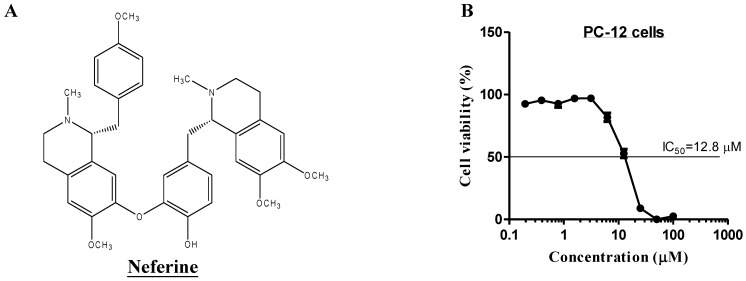

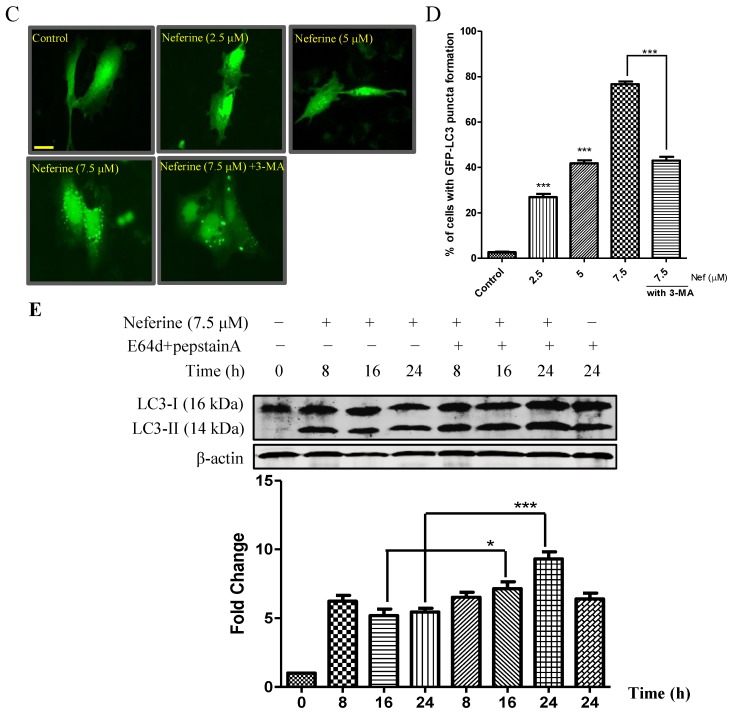
Neferine induces autophagy. (**A**) Chemical structure of neferine. (**B**) Cell viability was measured at 48 h after treatment with neferine. The IC_50_ value (12.8 µM) of neferine was determined graphically from the survival curve. (**C**) PC-12 cells transfected with GFP-LC3 plasmids were incubated with neferine (0 to 7.5 µM) with or without the presence of 3-methyladenine (3-MA, 5 mM) for 12 h. Representative images demonstrated that the number of LC3-positive cells was smaller after 3-MA treatment. Magnification: ×40. Scale bar: 15 µm. (**D**) Quantitation on the percentage of cells with GFP-LC3 puncta formation. (**E**) PC-12 cells were treated with neferine with or without the presence of E64d and pepstatin A (lysosomal protease inhibitors, 10 µg/mL) for 0 to 24 h. The level of LC3 II expression (14 kDa) was significantly higher after neferine treatment with the presence of lysosomal protease inhibitors. LC3-II band intensities were quantified using densitometric analysis and normalized to β-actin. Data are expressed as the fold change relative to the DMSO-treated negative control. Bars: SD. *** *p* < 0.001; * *p* < 0.05.

Neferine (Nef), a bisbenzylisoquinoline alkaloid isolated from the lotus seed embryo of *Nelumbo nucifera* Gaertn (Figure 1A), has been reported for its protective effect on endothelial cells via nitric oxide production [15], reversing multidrug resistance in cancer [16], as well as its anti-proliferative [17], antioxidant and anti-inflammatory capacities [18]. With the strong correlation between age-related neurodegeneration diseases and autophagy malfunction, we started by monitoring the autophagy activity of neferine by transiently expressing the green fluorescent protein microtubule-associated protein light chain 3 (GFP-LC3) [19] in PC-12 cells. Upon autophagy induction, the essential step of conversing cytosolic LC3-I (16 kDa) to membrane-bound LC3-II (14 kDa) was observed and quantified by immunofluorescence microscopy and immunoblotting, respectively [20]. To determine the optimal concentration of neferine required for the induction of autophagy, the IC_50_ value of neferine was determined graphically from the survival curve, as shown in Figure 1B. Neferine was then evaluated for its ability to induce the formation of GFP-LC3 puncta, a marker of autophagy, by using concentrations (0 to 7.5 µM) below its IC_50_ value (12.8 µM).

To begin, PC-12 cells with GFP-LC3 expression were incubated with neferine, and the percentage of cells that showed an increase in autophagosome formation (as represented by GFP-LC3 puncta formation) was monitored by immunofluorescence microscopy. As shown in Figure 1C,D, while 7.5 µM of neferine induced the highest percentage of cells with GFP-LC3 puncta formation, 5 µM and 2.5 µM of neferine showed a moderate autophagic effect after treatments. Furthermore, there was a significant reduction in GFP-LC3 puncta formation when cells were treated with the presence of the autophagy inhibitor (3-methyladenine, 3-MA), a specific inhibitor of the class III PI3K, which stops autophagy upon inhibition [21]. These data suggest the autophagic activity induced by neferine.

To further confirm its autophagic activity, PC-12 cells treated with neferine were analyzed by Western blot for LC3-I to LC3-II conversion. However, it should be noted that an increase in the LC3-II level could either result from an increase in LC3-II formation or be due to the failure of the fusion between autophagosomes and the lysosomes [22]. To differentiate between the two events, the level of LC3-II was assayed with the presence of two lysosomal inhibitors (E64d and pepstatin A), which block the fusion between autophagosomes and lysosomes [23,24]. As shown in Figure 1E, neferine increased the rate of LC3-II formation in the presence of protease inhibitors. These data suggest that neferine induces autophagy as a result of increased autophagosome formation, but not the failure of autolysosome formation.

However, our results demonstrated that the autophagic induction was associated with an upregulation of p62, which is a substrate for autophagic degradation [25] (Figure S1A). Real-time PCR analysis confirmed that the increased level of p62 was due to the upregulation of the transcription level of p62 mRNA (Figure S1B). Accordingly, inhibition of protein synthesis by actinomycin D led to a marked reduction in p62 protein in response to neferine treatment (Figure S1C), suggesting that p62 was eventually subjected to degradation by autophagy.

### 2.2. Neferine Induces Autophagy through the mTOR-AMPK-Dependent Pathway

**Figure 2:**
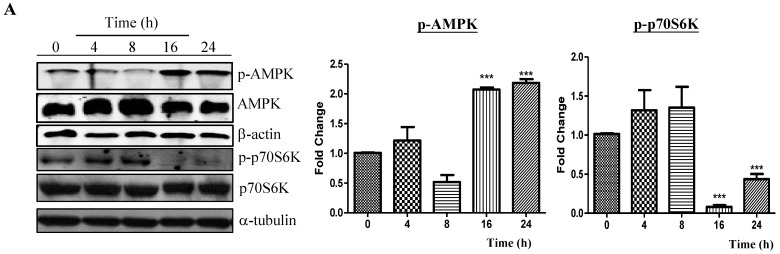

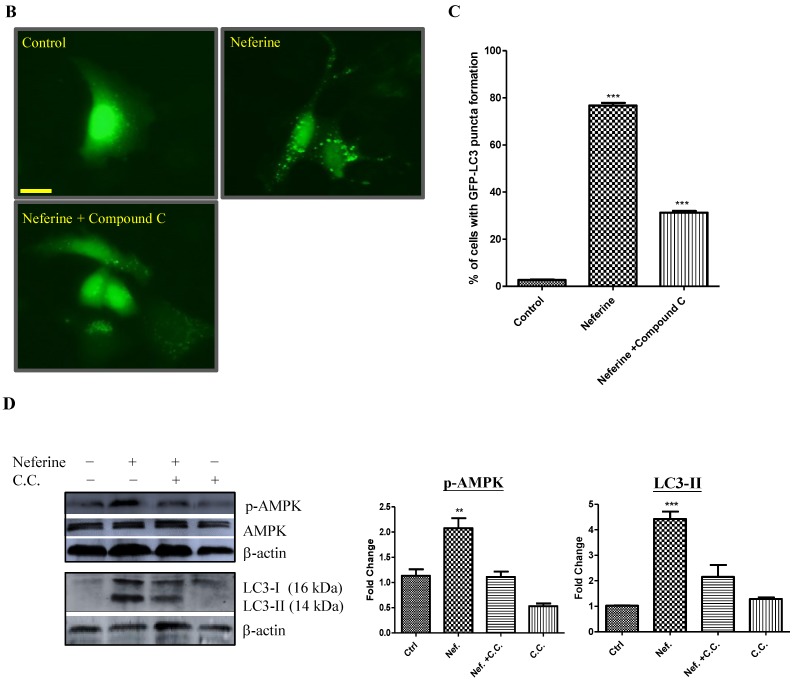
Neferine induces autophagy via activation of the AMPK-mTOR signaling pathway. (**A**) PC-12 cells were treated with neferine (7.5 µM) from 0–24 h. The increased level of phosphorylated AMPK was followed by a reduction in the phosphorylated level of p70S6K. Band intensities were quantified using densitometric analysis and normalized to β-actin or α-tubulin. (**B**) PC-12 cells transfected with GFP-LC3 were treated with 7.5 µM of neferine with the presence or absence of compound C (CC, 5 µM) for 12 h. Representative images demonstrated that the number of LC3-positive cells was smaller after compound C treatment. Magnification: ×40. Scale bar: 15 µm. (**C**) Bar chart representing the percentage of cells with GFP-LC3 puncta formation after treatments. Columns are the means of three independent experiments. (**D**) PC-12 cells were treated with neferine (7.5 µM) with the presence or absence of compound C (5 µM) for 24 h. The level of p-AMPK and LC3 II expression (14 kDa) was significantly lower after neferine treatment with the presence of compound C. Band intensities were quantified and normalized to β-actin. Data are expressed as the fold change relative to the DMSO-treated negative control. Bars: SD. *** *p* < 0.001; ** *p* < 0.01.

mTOR (mammalian target of rapamycin) kinase, an evolutionarily conserved protein kinase regulating autophagy, is the central regulator of cell growth and regulates autophagy in response to cellular conditions, such as starvation, growth factor deprivation and stress stimulation [26]. Furthermore, autophagy can be activated by AMP-activated protein kinase (AMPK), which acts as the key energy sensor for maintaining cellular metabolism and homeostasis [27]. It was reported that while AMPK promotes autophagy during starvation by directly activating Ulk1, the mammalian autophagy-initiating kinase, activation of mTOR could prevent Ulk1 activation and inhibit autophagy induction under nutrient sufficient conditions [26]. As shown in Figure 2A, PC-12 cells treated with neferine showed an increase in the phosphorylation of AMPK in a time-dependent manner. In addition, there was a significant reduction in GFP-LC3 puncta formation in cells treated with the presence of compound C (Figure 2B,C), which is a specific inhibitor of AMPK. The observation was further confirmed by a decrease in p-AMPK and the LC3-II level as revealed by Western blot analysis (Figure 2D). This result was further accompanied by a concomitant reduction in the 70-kDa ribosomal protein S6 kinase (p70S6K) phosphorylation (Figure 2A). p70S6K is a mitogen-activated serine/threonine kinase responsible for the control of cell growth and acts as the downstream target of mTOR kinase [28]. Taken together, these data suggest the possible signaling pathway of neferine, which may induce autophagy through an AMPK-mTOR-dependent manner.

### 2.3. Neferine Enhances the Clearance of Huntingtin Protein and Inclusion in an Atg7-Dependent Manner

**Figure 3:**
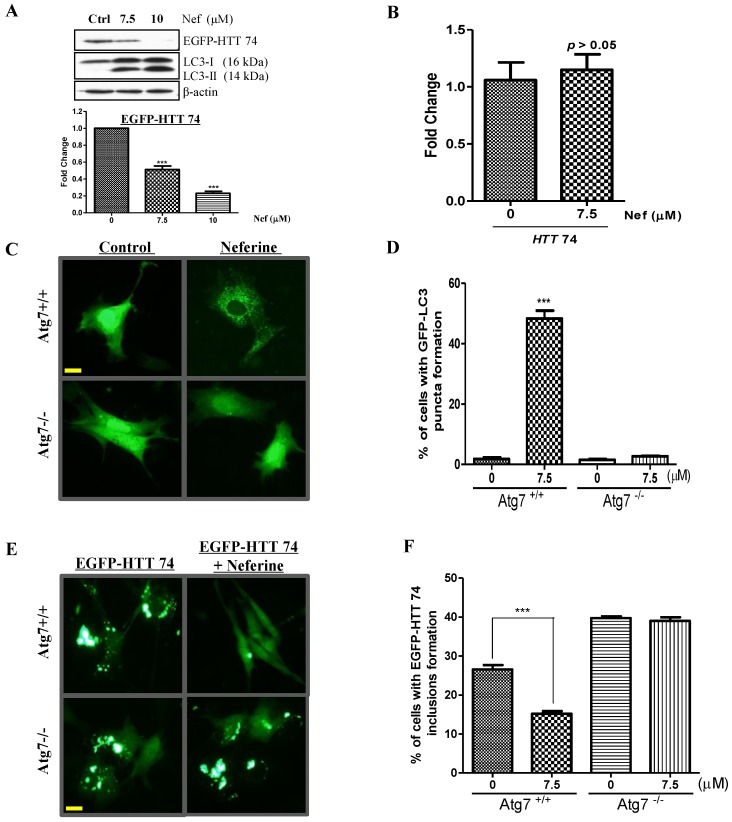

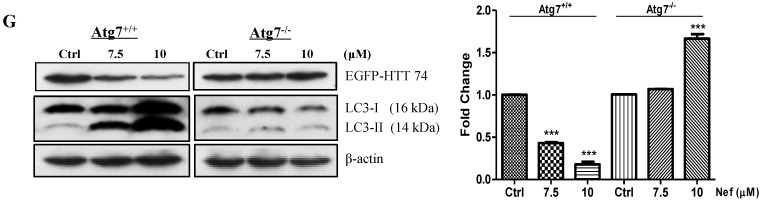
Neferine enhances the clearance of mutant huntingtin. (**A**) PC-12 cells transfected with EGFP-*HTT* 74 for 16 h were treated for 24 h with neferine at 7.5 µM and 10 µM, respectively. The protein level of mutant huntingtin decreased with an increased level of LC3-II after neferine treatments. EGFP-HTT 74 band intensities were quantified and normalized to β-actin. (**B**) PC-12 cells transfected with EGFP-*HTT* 74 were analyzed by real-time PCR on the *huntingtin* level after 24 h of neferine (7.5 µM) treatment. No significant change in the transcriptional level of *huntingtin* was observed after neferine treatment. (**C**) Wild-type Atg7 and Atg7-deficient mouse embryonic fibroblasts (MEFs) transfected with GFP-LC3 for 16 h were treated with neferine (7.5 µM) for 24 h. Representative images demonstrated that the number of LC3-positive cells was low in Atg7-deficient MEFs when compared with wild-type MEFs. (**D**) Bar chart indicating the percentage of cells with GFP-LC3 puncta formation after treatments. (**E**) Wild-type Atg7 and Atg7-deficient MEF cells transfected with EGFP-*HTT* 74 were treated with neferine (7.5 µM) for 24 h before being subjected to fluorescent microscopy analysis. Representative images revealed that the number of EGFP-HTT 74 inclusions was larger in Atg7-deficient MEFs when compared with wild-type MEFs. (**F**) Bar chart showing the percentage of cells with EGFP-HTT 74 inclusion formation. (**G**) Wild-type Atg7 and Atg7-deficient MEF cells transfected with EGFP-*HTT* 74 for 16 h were treated with 24 h of neferine (7.5 and 10 µM). The protein level of mutant huntingtin was reduced with an increased level of LC3-II in wild-type, but not Atg7-deficient MEFs. EGFP-HTT 74 band intensities were quantified and normalized to β-actin. Data are expressed as the fold change relative to the DMSO-treated, negative control. Bars: S.D. *** *p* < 0.001. Magnification: ×40. Scale bar: 15 µm. Columns are the means of three independent experiments. Ctrl, control.

Autophagy has been considered as a new therapeutic approach for modulating neurodegenerative disorders caused by the accumulation of aggregate-prone proteins, such as Parkinson’s disease (PD), Alzheimer’s disease (AD) and Huntington’s disease (HD) [29]. Huntington’s disease (HD), an autosomal-dominant neurodegenerative disease, is caused by a larger than 35-trinucleotide (CAG) repeat in huntingtin, which finally leads to the accumulation of abnormal long polyglutamine protein and toxicity [30]. As a potent autophagy inducer, we therefore investigate if neferine is able to enhance the clearance of mutant huntingtin through an autophagy-dependent pathway. To this end, we transiently overexpressed the mutant *huntingtin* with 74 CAG trinucleotide repeats (EGFP-*HTT* 74) in PC-12 cells. As shown in Figure 3A, neferine enhanced the clearance of overexpressed EGFP-tagged mutant huntingtin with a concomitant increased expression of LC3-II. Furthermore, real-time PCR results confirmed that neferine did not affect the transcriptional level of *huntingtin* in PC-12 cells (Figure 3B).

To further confirm that neferine accelerates the removal of mutant huntingtin through an autophagy-dependent manner, immunocytochemistry analysis using wild-type autophagy-related gene 7 (Atg7) and Atg7-deficient mouse embryonic fibroblasts (MEFs) were performed. During autophagy induction, LC3-I is activated by Atg7 and transferred to the Atg3. This is then followed by the conjugation of LC3-I to phosphatidylethanolamine (PE) [31], where LC3-I becomes membrane-bound LC-3-II and leads to autophagosome formation [32]. As shown in Figure 3C,D, neferine induced GFP-LC3 puncta formation in wild-type Atg7 cells, but not in Atg7-deficient MEFs, which are resistant to autophagy induction [33]. Furthermore, neferine enhanced the clearance of cytoplasmic EGFP-HTT 74 fluorescent inclusions in wild-type Atg7 cells, but not in Atg7-knockout cells transfected with EGFP-*HTT* 74 (Figure 3E,F). As further revealed by Western blot analysis, neferine enhanced the expression of LC3-II and the clearance of EGFP-tagged mutant huntingtin protein in wild-type, but not Atg7-deficient MEFs (Figure 3G). These data suggested that neferine works as a potent autophagy inducer, which accelerates the removal of mutant huntingtin inclusions and proteins through an autophagy gene (Atg7)-dependent mechanism.

### 2.4. Neferine Confers No Obvious Cytotoxicity at Its Effective Working Concentration

**Figure 4:**
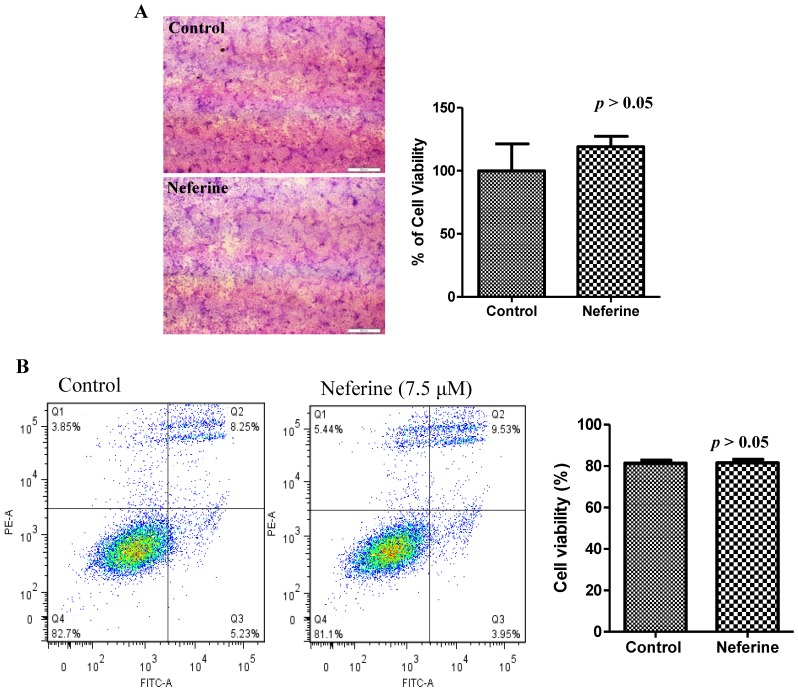

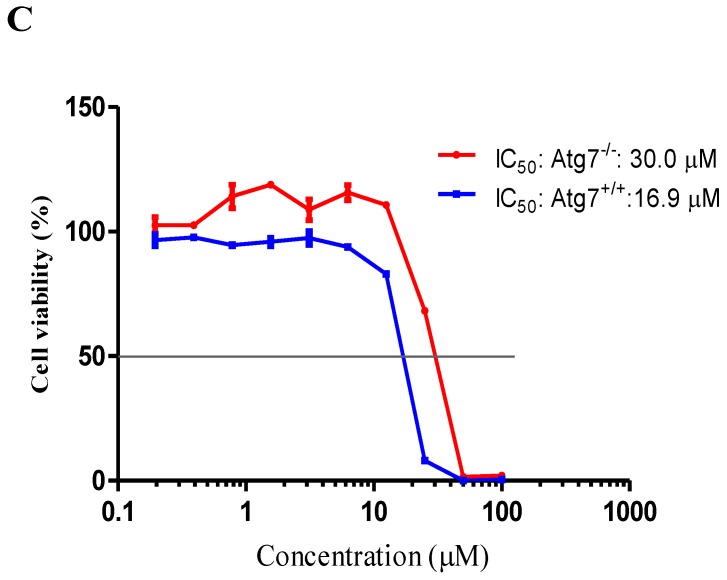
Neferine confers no obvious cytotoxicity at its effective working concentration. (**A**) PC-12 cells were stained with crystal violet for cell visualization after 24 h of neferine treatment (7.5 µM). Representative bright field images were captured (magnification: ×4). Scale bar: 150 µm. No significant difference in the percentage of cell viability was detected after measuring the absorbance (OD: 570 nm) of dissolved stained cells with or without the neferine treatments. (**B**) PC-12 cells were treated with neferine (7.5 µM) for 24 h. No significant difference in cell death was detected by annexin V staining using flow cytometry after neferine treatments. (**C**) Both wild-type Atg7 and Atg7-deficient MEF cells were treated with neferine from 0 to 100 µM for 48 h. The cytotoxicity (IC_50_ value) of neferine was then measured by the MTT assay. Data from the bar chart represent the means ± SD of three independent experiments.

To study the possibility of developing neferine into a potential therapeutic neuro-protective agent, we first demonstrated that neferine confers no obvious cytotoxicity at its effective working concentration (7.5 µM) in PC-12 cells by crystal violet (Figure 4A) and FITC-annexin V staining (Figure 4B). Furthermore, the cytotoxicity (IC_50_ value) of neferine against both wild-type Atg7 and Atg7-deficient MEF cells was also determined by the MTT assay (Figure 4C). 

### 2.5. Neferine Reduces the Toxicity of Mutant Huntingtin in Cells

**Figure 5:**
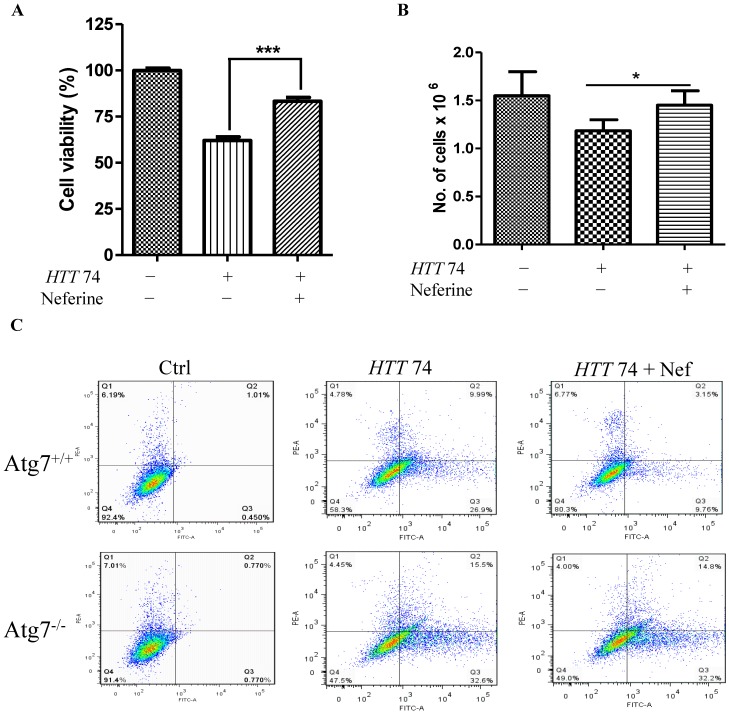

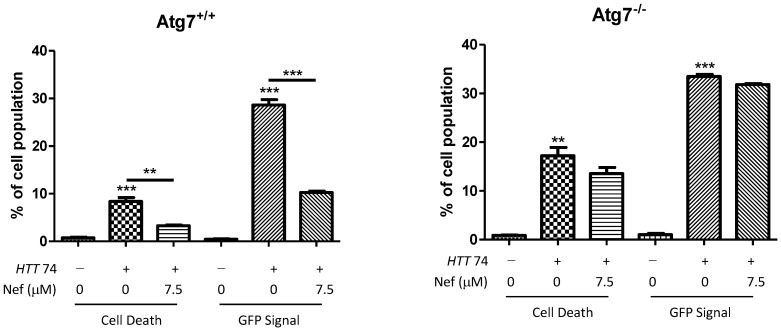
Neferine reduces the toxicity of mutant huntingtin in cells. (**A**) PC-12 cells transfected with *huntingtin* with 74 CAG repeats for 16 h were treated with neferine for a further 24 h. Cell viability was then measured by the MTT assay or (**B**) by counting the number of viable cells after treatments. A significantly higher percentage of cell viability was detected after neferine treatments. (**C**) Wild-type Atg7 and Atg7-deficient MEF cells transfected with *huntingtin* with 74 CAG repeats (EGFP-*HTT* 74) for 6 h were treated with neferine for a further 48 h. A significant lower percentage of cells with positive propidium iodide staining (cell death) and GFP signals was detected in wild-type Atg7 cells after neferine treatments. Data from the bar chart represent the means ± SD of three independent experiments. * *p* < 0.05; ** *p* < 0.01; *** *p* < 0.001.

In addition to the accumulation of abnormal long polyglutamine huntingtin protein in cells, mutant huntingtin confers toxicity in cells when it is cleaved to form the N-terminal fragments, which contain the first 100–150 residues with the expanded polyglutamine [34]. Therefore, we have further investigated the protective role of neferine on the toxicity of mutant huntingtin in cells. With the result that neferine enhanced the clearance of mutant huntingtin *in vitro*, we overexpressed PC-12 cells with EGFP-*HTT* 74 and then studied the effect of neferine on mutant huntingtin-induced cell death. As shown by the results of the MTT assay (Figure 5A), while transient expression of mutant huntingtin leads to a decrease in cell viability, neferine attenuated cell death induced by mutant huntingtin. However, as the MTT assay is a metabolic assay that measures mitochondrial activity, in order to exclude the possibility that neferine may have a direct effect on mitochondria, we further performed the measurement of cell viability on neferine-treated PC-12 cells, by directly counting the number of viable cells after treatments. As shown in Figure 5B, neferine rescued PC-12 cells from mutant huntingtin-induced cell death, which further confirmed the protective role of neferine on mutant protein-induced cell death. In Figure 5C, the protective effect of neferine on mutant huntingtin-induced cell death was elucidated in both wild-type and Atg7-deficient MEF cells. As revealed by the percentage of cells with a positive GFP (FITC) signal, the percentage of GFP-positive cells with the overexpression of mutant huntingtin was significantly lower in wild-type MEF cells compared to Atg7-deficient MEF cells after treatment with neferine. Concomitantly, the percentage of cell death was lower in wild-type MEF cells than in Atg7-deficient MEF cells after neferine treatments. Consistent with our previous findings that neferine enhanced the clearance of mutant huntingtin possibly through an Atg7-dependent mechanism, our results therefore supported the potential therapeutic role of neferine working as a neuroprotective agent, which reduced cell death induced by mutant huntingtin at the cellular level.

## 3. Experimental Section

### 3.1. Reagents, Chemicals, Antibodies and Plasmids

All chemicals and reagents were purchased from Sigma-Aldrich (St. Louis, MO, USA) unless otherwise specified. Neferine (>98% purity, HPLC) was purchased from Chengdu MUST Bio-technology Company Ltd. (Chengdu, China). Compound C (CC) was obtained from Calbiochem (San Diego, CA, USA). EGFP-*HTT* 74 plasmid was a generous gift from David C. Rubinsztein (University of Cambridge, Cambridge, UK). pEGFP-LC3 reporter plasmid was kindly provided by Tamotsu Yoshimori (Osaka University, Osaka, Japan). Monoclonal antibodies (1:1000) against LC3B (#3868s), p-AMPK (Thr172) (#2535) or AMPK (#2532) and polyclonal antibodies (1:1000) against p-p70S6K (Thr389) (#9205) or p70S6K (#9202) were purchased from Cell Signaling Technologies Inc. (Beverly, MA, USA). Monoclonal anti-GFP (sc-9996) and -p62 (sc-28359) antibodies were purchased from Santa Cruz Biotechnology (Santa Cruz, CA, USA). Monoclonal β-actin (A2228) and α-tubulin (T9026) antibodies were purchased from Sigma (St. Louis, MO, USA).

### 3.2. Cell Culture

All cells were obtained from the American Type Culture Collection (ATCC) (Rockville, MD, USA), unless otherwise stated. PC-12 cells were cultured with F-12K medium supplemented with 10% horse serum, 50 U/mL penicillin and 50 µg/mL streptomycin (Invitrogen, Paisley, Scotland, UK) in a humidified incubator at 37 °C with 5% CO_2_. Wild-type Atg7 and Atg7-deficient mouse embryonic fibroblasts (MEF), provided by Masaaki Komatsu (Juntendo University, School of Medicine, Tokyo, Japan), were supplemented in DMEM with 10% fetal bovine serum. 

### 3.3. Quantification of GFP-LC3 Puncta Formation

GFP-LC3 puncta formation was monitored and quantified as described in [20]. In brief, cells were grown inside a 6-well plate with coverslips. After neferine treatments, cells were fixed with 4% paraformaldehyde for 20 min. Before being subjected to fluorescence microscopic analysis, coverslips with cells were mounted with FluorSave™ mounting media (Calbiochem, San Diego, CA, USA). The number of GFP-positive cells with GFP-LC3 puncta formation was examined under the Nikon ECLIPSE 80i microscope. Representative images were captured with the CCD digital camera Spot RT3™ (Diagnostic Instruments, Inc., Melville, NY, USA). To quantify autophagy, the percentage of cells with punctate GFP-LC3 fluorescence was calculated by counting the number of cells with punctate GFP-LC3 fluorescence in GFP-positive cells. A minimum of 200 cells from 3 randomly selected fields was scored.

### 3.4. Cytotoxicity Assays and Flow Cytometry

Cell viability was measured by using the MTT method (3-[4,5-dimethylthiazol-2-yl]-2,5-diphenyl tetrazolium bromide) as described in [20]. Besides, the viability of cells was measured by crystal violet staining after neferine treatments. This is performed by incubating PC-12 cells with 7.5 µM of neferine for 24 h. The cells were then stained with crystal violet for 10 min and then rinsed gently by distilled water. The stained bright field cell image was captured by the CCD digital camera Spot RT3™ under the Nikon ECLIPSE 80i microscope (4× magnification). Cell viability was further quantified by dissolving stained cells in 10% acetic acid [32]. Colorimetric reading was then measured by spectrophotometer at OD 570 nm. The percentage of cell viability was calculated using the following formula: cell viability (%) = cells number_treated_/cells number_DMSO control_ × 100. Data were obtained from three independent experiments.

Cell viability was also determined by using an annexin V staining kit (BD Biosciences, San Jose, CA, USA). In brief, PC-12 cells treated with 7.5 µM of neferine were harvested and analyzed by multiparametric flow cytometry using FITC-annexin V and/or propidium iodide staining (BD Biosciences, San Jose, CA, USA), according to the manufacturer’s instructions. Flow cytometry was then carried out using a FACSCalibur flow cytometer (BD Biosciences). Data acquisition and analysis were performed with CellQuest (BD Biosciences).

### 3.5. Western Blot Analysis

Cells were harvested and lysed with RIPA buffer (Cell Signaling Technologies, Beverly, MA, USA) after treatments. Protein concentrations were quantitated by using Bradford reagent (Bio-Rad, Hercules, CA, USA) before electrophoresis. After electrophoresis, proteins were transferred to the membrane, which was then blocked with 5% non-fat dried milk. Corresponding primary antibodies were incubated with the membrane overnight at 4 °C with constant shaking. The membrane was then incubated with HRP-conjugated secondary antibodies before being visualized by using the ECL Western Blotting Detection Reagents (Invitrogen, Paisley, Scotland). Band intensities were quantified by using the software, ImageJ (ImageJ 1.46r; National Institutes of Health, Bethesda, MD, USA).

### 3.6. Detection of Mutant Huntingtin Protein and Inclusions 

To study the neuro-protective effects of neferine, cells were transiently transfected with EGFP-*HTT* 74 plasmids by using Lipofectamine Plus LTX reagent (Invitrogen, Paisley, Scotland, UK). The cells with mutant huntingtin expression were then treated with neferine for 24 h. The protein level of the mutant huntingtin (EGFP-HTT 74) was quantitated by immunoblotting with antibody against GFP. Wild-type Atg7 and Atg7-deficient MEF cells transfected with EGFP-*HTT* 74 were subjected to fluorescent microscopy analysis for cytoplasmic huntingtin inclusion formation after 24 h of neferine treatment. Percentages of cells with EGFP-HTT 74 inclusion formation were counted by the number of cells with EGFP inclusions over the total number of EGFP-positive cells in the same field. More than 200 GFP-positive cells were scored for each treatment. 

### 3.7. Real-Time PCR Analysis

PC-12 cells transfected with EGFP-*HTT* 74 were incubated with neferine for 24 h. Total RNA was then extracted from the cells by using the FavorPrep™ Total RNA purification mini kit (Favorgen, PingTung, Taiwan). cDNA was synthesized by performing reverse transcription using SuperScript^®^ VILO™ Master Mix (Invitrogen, Scotland, UK). FS Universal SYBR Green Master Rox (Roche, Indianapolis, IN, USA) was used according to the manufacturer’s instructions. Primers 5'-ATG AAG GCC TTC GAG TCC CTC AAG TCC TTC-3' and 5'-GGC GGC TGA GGA AGC TGA GAA-3' were used as described in [35]. All of the real-time PCRs were carried out on the ViiA™ 7 Real Time PCR System (Applied Biosytems, Grand Island, NY, USA).

### 3.8. Statistical Analysis

The results were expressed as means ± SD, as indicated. The difference was considered statistically significant when the *p*-value was less than 0.05. Student’s *t*-test or one-way ANOVA analysis was used for comparison among different groups.

## 4. Discussion

Neurodegenerative diseases, such as Huntington disease (HD), Parkinson disease (PD) and Alzheimer’s disease, are caused by toxic, aggregate-prone or oligomeric proteins [6,8,13]. These mutant proteins are highly associated with aggregate formation and toxicity [11,34]. Some mutant proteins, such as A53T/A30P α-synuclein or huntingtin, are identified as substrates of autophagy and can be degraded by autophagy [2,6,8,34,36]. While small cytosolic proteins are degraded by proteasomes, large membrane proteins, oligomers and aggregates, which cannot pass through the narrow pore of the proteasome barrel, are degraded by autophagy [30,37]. It was shown that pharmacological activation of autophagy reduces the levels and toxicity of mutant huntingtin, mutant proteins in spinocerebellar ataxia, mutant α-synuclein and mutant tau in either mouse or *Drosophila* models [13,38]. In addition, inositol hexakisphosphate kinase type 2 (InsP6K2) was reported to induce autophagy, a protein aggregate clearance process in HD patients, and apoptotic cell death concomitantly, possibly via the Akt/phosphatidylinositol 1,4,5-trisphosphate pathway in HD lymphoblast or mutant *HTT* transfected cells [39,40]. These findings further supported that the pathogenesis of HD is highly related to the accumulation of toxic proteins or aggregates, which lead to the induction of autophagy, which could facilitate the removal of protein aggregates in cells. 

Recent reports have demonstrated that trinucleotide expansions lead to disease by both protein- or RNA-regulated mechanisms. Besides, CAG expansion constructs could lead to expression of polyglutamine proteins in the absence of an ATG start codon (repeat-associated non-ATG translation, RAN translation). This toxic and unexpected RAN protein translation are reported in the pathogenesis of microsatellite disorders, such as spinocerebellar ataxia type 8, myotonic dystrophy type 1, fragile X tremor ataxia syndrome and frontotemporal dementia [41]. Recent analysis demonstrated that RAN translation occurs in all reading frames, even with the presence of the ATG-codon open reading frame (ORF) [41], leading to the production of homopolymeric toxic proteins. Therefore, with the protective autophagic effect in attenuating the level and toxicity of mutant proteins induced through the ATG-codon-regulated expression of mutant *huntingtin* in PC-12 cells, neferine may also play a protective role in facilitating the removal of RAN proteins via the induction of autophagy. Hence, it worth further exploring the potential protective role of neferine in non-canonical RAN translation of HD, as well as in other non-canonical RAN translation-related microsatellite diseases [42], via the induction of autophagy. 

Active compounds isolated from Chinese medicinal plants were found to be effective at modulating neurodegenerative disorders [43]. For example, salidroside can protect against amyloid-β peptide (Aβ)-induced oxidative damage in PC-12 cells [44]. Curcumin acts as an effective agent against amyloid beta-aggregation [45]. Huperzine A is able to reduce glutamate-induced toxicity in neurons [46]. Recently, we have identified that onjisaponin B derived from *Radix Polygalae* is able to enhance autophagy and accelerates the degradation of mutant α-synuclein and huntingtin in cells [32]. Therefore, the identification of new chemical entities from Chinese medicinal herbs for neuroprotective functions becomes an important means of novel drug discovery. 

A recent study suggested that neferine can induce autophagy, ROS hyper-generation, PI3K/Akt/mTOR inhibition and cytotoxicity in lung cancer cells [17]; however, the role of PI3K/AKT/mTOR inhibition on autophagy induction, as well as on the neferine-mediated cytotoxicity remains unclear. In contrast, by using the AMPK inhibitor (compound C), our present study reports for the first time that neferine, a small-molecule autophagy inducer, may act as a potential neuro-protective agent at its sub-lethal dose, through activating the AMPK-mTOR pathway. In addition, by using the autophagy-deficient cellular model, we confirmed that the autophagy effect of neferine was Atg7 dependent. As demonstrated by a significant increase in mutant huntingtin aggregation and toxicity in Atg7-deficient cells, as compared with wild-type Atg7 cells after neferine treatments, our results further suggested the novel protective role of neferine-mediated autophagy in reducing mutant huntingtin aggregation and toxicity in cells. 

For instance, rapamycin, an inducer of mammalian target of rapamycin (mTOR)-dependent autophagy, increases autophagic clearance of mutant huntingtin fragments *in vivo* [6,13]. Working as a similar molecular pathway as rapamycin, our results demonstrated that the neferine-mediated autophagy induction is dependent on the AMPK-mTOR pathway. Although mTOR inhibition has adverse effects in protein synthesis and cell proliferation [1,14], with the advantage that neferine confers no obvious cytotoxicity at its effective concentration, neferine may work as a useful neuro-protective agent through effective degradation of mutant proteins. Finally, although neferine may play a neuroprotective role in the cellular neurodegenerative disease model, the association between its autophagic activity and neuroprotective mechanism remains to be elucidated by further *in vitro* and *in vivo* disease models in the future.

## Supplementary Materials

Supplementary materials can be accessed at: http://www.mdpi.com/1420-3049/20/03/3496/s1.
