# Supplementary material for: Neferine Attenuates the Protein Level and Toxicity of Mutant Huntingtin in PC-12 Cells via Induction of Autophagy
Source: Molecules. 2015 Feb 18;20(3):3496–514. doi: 10.3390/molecules20033496 (PMC6272412; doi:10.3390/molecules20033496)
Supplement: Supplementary file 1 [file molecules-20-03496-s001.pdf]

Supplementary Materials

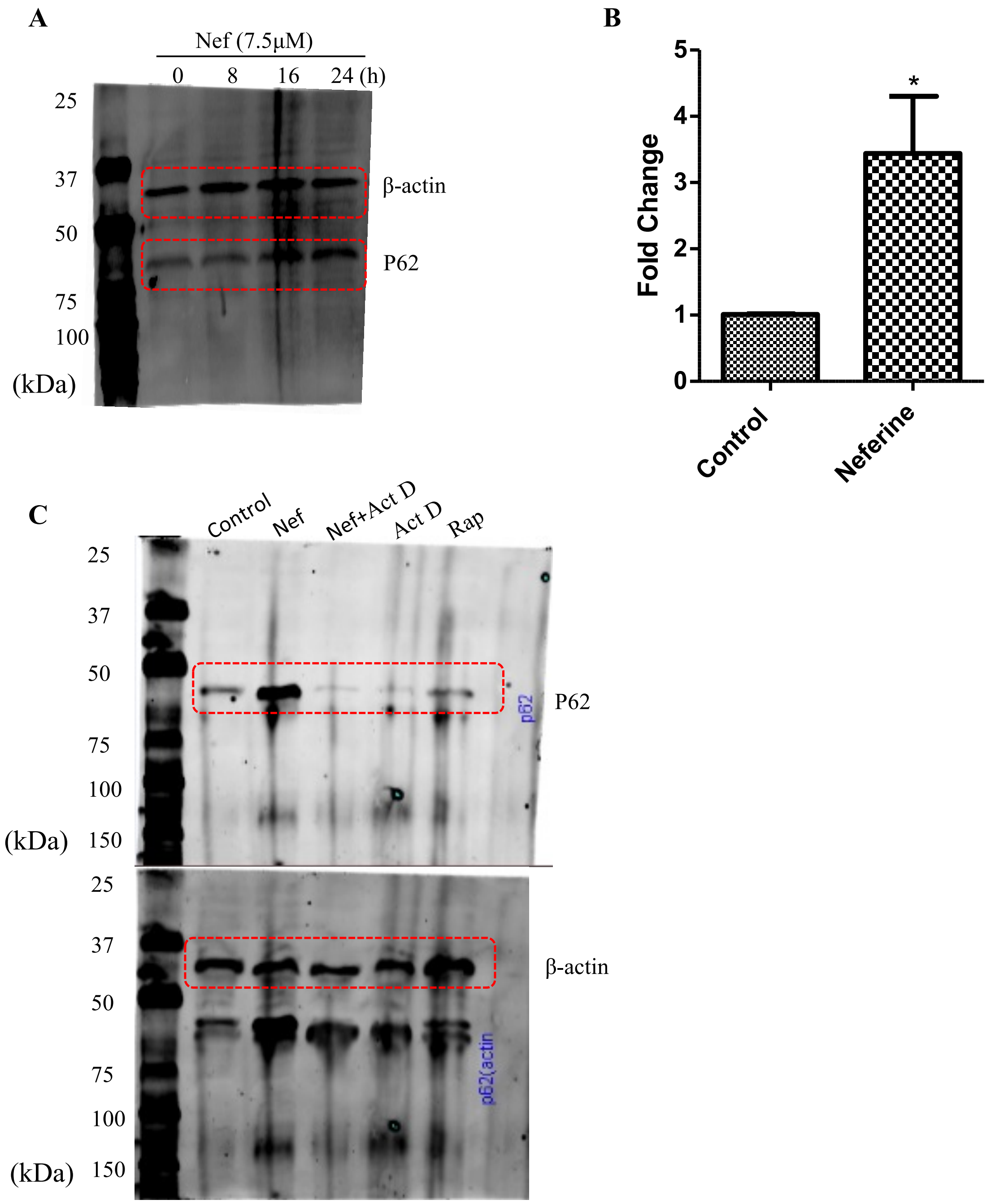

Figure S1. Neferine up-regulates the mRNA and protein level of p62. (**A**) Full-length immunoblot of SDS-PAGE showing the protein levels of p62 from 0–24 h of neferine treatment (7.5 mM). (**B**) PC-12 cells were analyzed by real-time PCR on the p62 mRNA level and normalized against the housekeeping gene (β-actin) after 24 h of neferine (7.5 mM) treatment. (**C**) PC-12 cells were pre-treated with actinomycin D (ACD) (2.5 mg/mL) for 1 h before neferine (7.5 μM) treatment for 0–16 h. Cells treated with 0.3 mM of rapamycin (Rap) for 24 h were used as the positive control. Cell lysates were then analyzed for p62 and β-actin. Columns, means of three independent experiments; bars, SEM. * $p < 0.01$.
